# X-ray Zernike phase contrast tomography: 3D ROI visualization of mm-sized mice organ tissues down to sub-cellular components

**DOI:** 10.1364/BOE.396695

**Published:** 2020-09-10

**Authors:** E. Longo, L. Sancey, S. Flenner, A. Kubec, A. Bonnin, C. David, M. Müller, I. Greving

**Affiliations:** 1 Helmholtz-Zentrum Geesthacht, Institute of Material Research, Max-Planck-Strasse 1, 21502 Geesthacht, Germany; 2 Institute for Advanced Biosciences U1209 UMR5309 UGA, Allée des Alpes - Site Santé, La Tronche, 38700, France; 3 Paul Scherrer Institut, Villigen PSI, 5232, Switzerland

## Abstract

Thanks to its non-invasive nature, X-ray phase contrast tomography is a very versatile imaging tool for biomedical studies. In contrast, histology is a well-established method, though having its limitations: it requires extensive sample preparation and it is quite time consuming. Therefore, the development of nano-imaging techniques for studying anatomic details at the cellular level is gaining more and more importance. In this article, full field transmission X-ray nanotomography is used in combination with Zernike phase contrast to image millimeter sized unstained tissue samples at high spatial resolution. The regions of interest (ROI) scans of different tissues were obtained from mouse kidney, spleen and mammalian carcinoma. Thanks to the relatively large field of view and effective pixel sizes down to 36 nm, this 3D approach enabled the visualization of the specific morphology of each tissue type without staining or complex sample preparation. As a proof of concept technique, we show that the high-quality images even permitted the 3D segmentation of multiple structures down to a sub-cellular level. Using stitching techniques, volumes larger than the field of view are accessible. This method can lead to a deeper understanding of the organs’ nano-anatomy, filling the resolution gap between histology and transmission electron microscopy.

## 1. Introduction

X-ray tomography is a high-resolution 3D imaging approach suitable for exploring the inner structure of different kind of materials, ranging from engineering to life sciences applications. Biological samples normally suffer from weak absorption contrast in the hard X-ray regime. Hard X-ray tomography is often combined with phase contrast techniques like free-space propagation [1], in-line holography [2], grating interferometers [3], and Zernike phase contrast [4–7] in order to study structural features in soft tissues [8]. Tomography has the great advantage of collecting 3D information by preserving the native state of the samples. Thanks to its non-destructive nature and the high brilliance offered by synchrotron facilities, X-ray tomography demonstrated to be a powerful tool for assessing the anatomy of human and rodent samples in several pre-clinical studies [9]. However, standard X-ray tomography (i.e. X-ray microtomography) is limited by a spatial resolution of the order of 0 - 1 µm. Thus, it is not appropriate for visualizing details at the cellular scale. In order to gain a deeper understanding of the tissues’ sub-architecture, X-ray nanotomography has to be applied. A range of studies has been published focusing on holotomography. Kimchenko et al. were one of the first groups performing nano-imaging of unstained human brain tissues with a spatial resolution down to 88 nm [10]. Töpperwien et al. demonstrated that nano-holotomography can complement histological inspections [11] and provide quantitative and statistical analysis with a half-period resolution of 370 nm [12]. Pacureanu et al. disentangled dense neuronal structures in Drosophila melanogaster and mice nervous tissue with measured resolutions from 222 nm down to 87 nm [13]. Massimi et al. explored the amyloid plaques morphology in mouse models of the Alzheimer’s disease [14] and Cedola et al. showed the degenerative effects of multiple sclerosis at the level of vascular and neuronal networks with a spatial resolution of 260 nm [15]. Though holotomography is a multi-scale approach bridging the gap between X-ray microtomography and nanotomography, it requires time consuming and computing intense reconstruction algorithms. In a typical holotomography configuration, tomographies have to be performed at 3 - 4 propagation distances for each sample for retrieving the quantitative phase signal. Moreover, specific algorithms based on the Fresnel diffraction model need to be applied prior to tomographic reconstruction in order to retrieve the phase shift caused by the sample [16]. Other nano-imaging studies were carried out by laboratory benchtop devices [17] or storage ring-based X-ray microscopes [18] following tissue staining protocols.

Zernike phase contrast enables fast, single distance nanotomography scans without the need of staining. In contrast to holotomography, Zernike reconstructions are fast and can even be performed on the fly. Even if the phase shift is not acquired in a quantitative way, the contrast enhancement is sufficient for the structural analysis of many biomedical and biological applications. Most of the transmission X-ray microscope (TXM) instruments using Fresnel Zone Plates (FZP) offer fields of view (FoVs) smaller than 50 µm x 50 µm in absorption and Zernike phase contrast mode [19–21]. Therefore ultra-small samples (between 8 - 100 µm in diameter) are required for imaging, prepared by focused ion beam scanning electron microscopy (FIB-SEM) [21,22].

This article is the first one to demonstrate that high-quality ROI nanotomography scans (in terms of contrast and resolution) can be obtained from millimeter sized tissue samples, without thinning or staining of the specimens. A large FoV was obtained using a beamshaper specifically designed for the presented TXM (see section 2), having 100 µm sub-fields, instead of the typically used 50 µm sub-fields [19,21]. The FoV can even be enlarged using stitching methods [19]. The 3D assessment and characterization of several tissues using this approach is presented here. The technique is compatible with the classical 2D histological analysis but with the advantage of handling 3D information without physical sectioning of the specimens and without using staining agents for enhancing the visualization of the tissue components. Compared to well-established light microscopes techniques enabling the 3D virtual histology, such as confocal microscopy, two-photon-microscopy, light-sheet microscopy [23–28] and nonlinear microscopy [29], X-ray Zernike nanotomography ensures a deep tissue penetration without extensive sample preparation (optical clearing solvents, fluorescence dyes, etc.). Zernike phase contrast allows the visualization of multiple anatomical features enabling spatial resolutions down to the nanometer scale and fast volumetric reconstructions. The achieved contrast and resolution permitted the identification of structural details down to sub-cellular components. Unlike soft X-ray tomography [30–32], the high photon energy permits imaging at room temperature and atmospheric pressure. Therefore the herewith presented technique can be considered as a powerful tool accessing samples of large volumes at the nanoscale, by complementing histological analysis and filling the resolution gap between histology and TEM.

## 2. Methods

### 2.1 Mice sample preparation

The experiment has been approved by the local and the national ethics committees, following the European guidelines for the use of animals (APAFIS #8782-201732813328550 v1). Mice were sub-cutaneously injected with 1M mice mammalian carcinoma cells, 2 weeks before euthanasia and after deep isoflurane induction. The kidneys, spleen and tumor were collected, dehydrated in ethanol and embedded in paraffin for long-term storage. Then, small samples of organs were isolated and glued on the top of pins to perform the X-ray imaging.

### 2.2 X-ray Zernike nanotomography setup

**Fig. 1.**
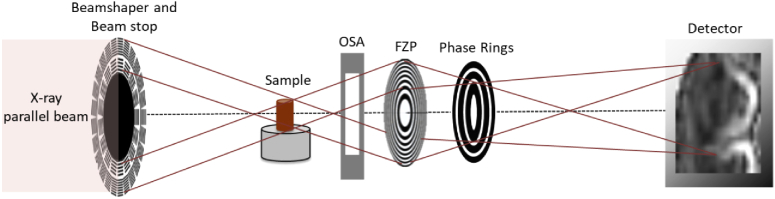
Schematic drawing of the Zernike TXM installed at the P05 imaging beamline, at PETRA III (DESY, Germany). The components "OSA" and "FZP" are the short abbreviation for "order sorting apertures" and "Fresnel Zone Plate", respectively.

The data were acquired at the P05 imaging beamline [33], operated by the Helmholtz-Zentrum Geesthacht (HZG), at the PETRA III storage ring at DESY in Hamburg, Germany. Different mice tissues were imaged by a full field high-resolution X-ray Zernike microscope, optimized for an energy of 11.1 keV [34]. The experimental setup (see Fig. 1) was composed of a beam-shaping condenser lens [35] for illuminating the sample, a Fresnel Zone Plate (FZP) for magnifying the sample image onto the detector and a set of concentric phase rings for achieving the Zernike phase contrast. The beam-shaping optics of 1.8 mm diameter and with 100 µm sub-fields produced a 100 µm x 100 µm square illumination at the sample plane. The FZP of 280 µm in diameter and 50 nm outermost zone was used as an objective lens. The FZP and the negative phase rings were made of gold and produced by electron-beam lithography and electroplating on silicon nitride membranes. The optimal working distance between sample and beam-shaper was 78 cm, while the phase rings were placed in the back focal plane of the FZP, i. e. 125.34 mm. The adopted configuration allowed achieving a FoV of 74 µm x 74 µm and an effective pixel size of 36 nm (binning 2 used, effective pixel size 72 nm). In order to block the direct beam, a beam stop with 800 µm diameter was installed close to the beam shaper; while four order sorting apertures were used to block higher orders. Illumination optics, FZP and phase rings were designed and fabricated by the Paul Scherrer Institut in Villigen, Switzerland. The raw images were acquired with a Hamamatsu X-ray sCMOS camera having a 10 µm Gadolinium oxysulfide scintillator, 2048 x 2048 number of pixels with a physical pixel size of 6.5 µm x 6.5 µm. 50 flat-field projections were acquired at the beginning and at the end of each tomographic scan and 100 dark images were used for the normalization. Each tomography was acquired in less than 30 minutes.

### 2.3 Data processing

In order to attenuate the noise and improve the density resolution [36], binning 2 was applied on the raw projections prior to the reconstruction; thus, leading to an effective pixel size of 72 nm. Subsequently, a median filter with a radius of 2 pixels was applied over the normalized projections. Moreover, for minimizing the ring artefacts, potential horizontal stripes were removed from the sinograms by applying the Fourier-wavelet method [37]. Tomographic image reconstructions were performed using the gridrec algorithm from tomopy [38,39]. The reconstructed volumes were analyzed with Fiji [40] image-processing package. The experimental resolution was estimated through the Fourier Shell Correlation with a half bit-threshold method [41,42], while the stitching was performed by the NRStitcher software [43] (Visualization 1). The 3D visualization of the data and the segmentation was achieved by Avizo Lite 9.4.

### 2.4 Histological analysis

Paraffin sections of 4 to 6 µm were obtained from the samples, de-paraffined using toluene, re-hydrated and staining with hematoxylin and eosin solutions. After staining, the samples were dehydrated, mounted, and imaged using a 10x microscope.

## 3. Results

In this work, full field transmission X-ray nanotomography (TXM) operated in Zernike phase contrast microscopy mode was used for a qualitative investigation of different mice organ tissues of macroscopic size (0.5 - 1mm). A representative scheme illustrating the adopted configuration is shown in Fig. 1.

This microscopy setup is currently available at the P05 imaging beamline (PETRA III, DESY) operated by the Helmholtz-Zentrum Geesthacht. This kind of arrangement was first described in Vartiainen et al. [35]. It consists of the following diffractive elements: a beamshaping optics for condensing the X-ray radiation onto the specimen and a Fresnel Zone Plate (FZP) lens for producing a magnified image of the sample onto the detector. In order to block the direct beam and the high orders, a beam stop is mounted downstream of the beamshaper and order sorting apertures (OSA) are installed downstream of the sample. The comparably large FoV of 74 µm x 74µm is achieved by using a beamshaper with 100 µm sub-fields (see section 2). The key element for Zernike phase contrast is a set of concentric phase rings placed in the back-focal plane of the FZP. The phase rings induce a phase shift of the background wave (undeviated by the sample) by pi/2. As a consequence, it interferes with the wave carrying the information about the sample structure and leads to phase-enhanced contrast imaged by the detector [44]. For the results shown in this work, negative phase rings were used that produced a background shift of 270 degree. By exploiting a long sample-detector distance (20.45 m), a magnification of 161 was achieved. This configuration allowed acquiring images with an effective pixel size of 36 nm and enabled accessing data with an experimental half-period resolution equal to 152.11 nm (see section 2), compared to the theoretical one, i.e. Rayleigh resolution [44], equal to 61 nm (i.e. 1.22 times the FZP zone width, 50 nm).

**Fig. 2.**
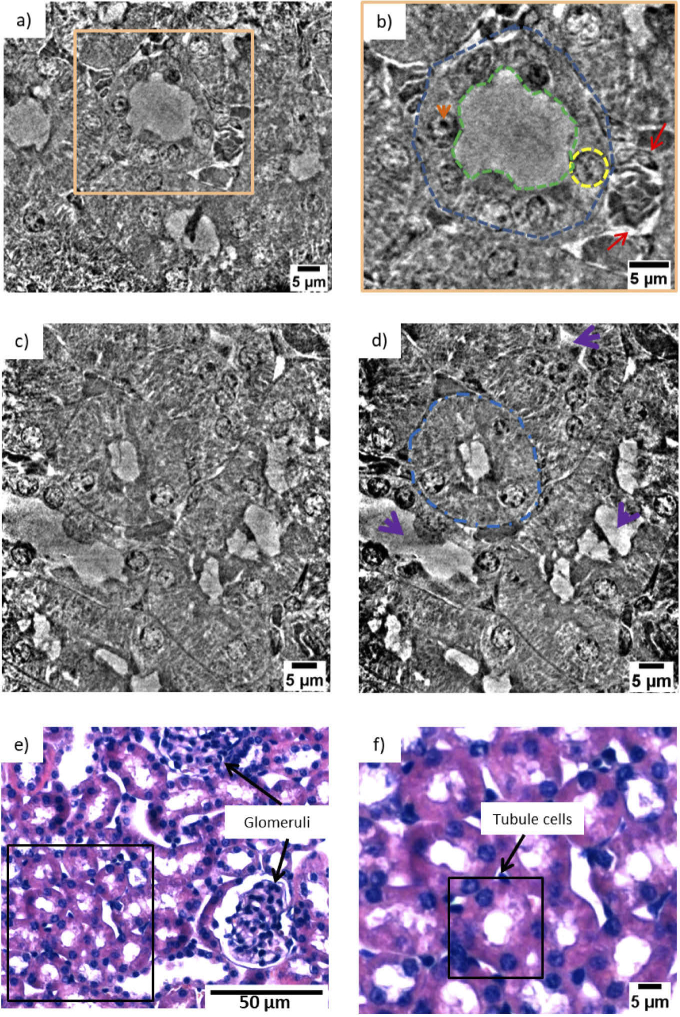
(a) Virtual nanotomography slice of the mouse kidney. (b) Zoomed view over one tubule cells group. The tubular structure is indicated by the blue dashed line, the lumen by the green dashed line and the nuclei by the yellow one. The orange arrow points at the nucleolus inside the nucleus. The two red arrows indicate a structure similar to a glomerulus. (c) Virtual tomographic slice enhancing tubule cells of different shape. (d) Minimum intensity projection image showing a distal tubule (blue dashed lines) surrounded by three proximal tubules (purple arrows). (e) Histological slice showing the kidney cortex anatomy. (f) Magnified view of the region within the black rectangle in (e).

The nano-anatomy of three different types of mice tissues were explored: kidney, spleen and mammalian carcinoma. All the imaged tissue samples had a macroscopic size ranging between 0.5 - 1 mm. The samples were extracted from paraffin-embedded organ slices of 1 mm thickness. Figure 2(a) shows a virtual slice of the kidney resulting from tomographic reconstruction. The image resolves the arrangement of several renal tubules and allows the identification of tubular ultra-structures in the range of 0.5 - 5 µm in size. The orange rectangle in Fig. 2(a) outlines a single tubule and the corresponding magnified image is displayed in Fig. 2(b). According to the grey-scale map, high-density regions are visualized in black and low-density regions are displayed in white. The tubules are nephron segments and are composed of different cells. The achieved contrast and resolution enabled the discrimination of the different elements composing the tubule cells.

**Fig. 3.**
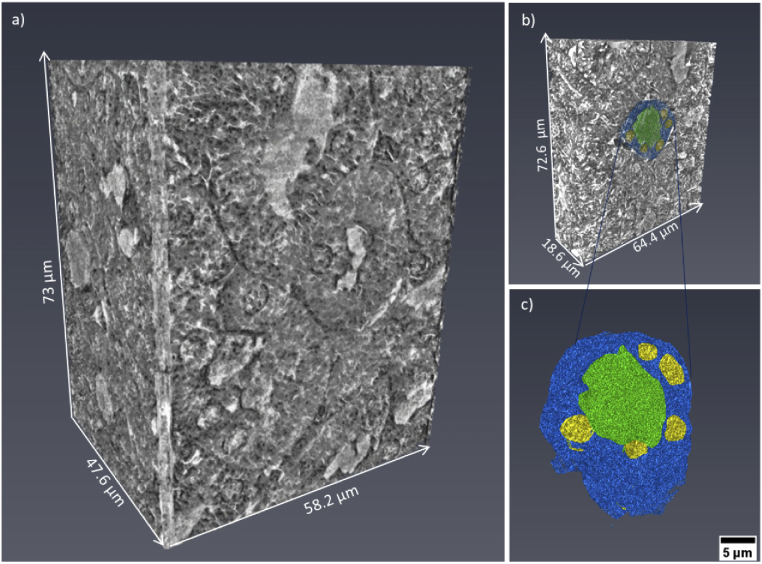
(a) 3D volume rendering in grey-scale of a volume of interest derived from the kidney tomographic dataset. (b) 3D rendering with segmented tubule cells. (c) Rendering of the 3D segmented tubule cells and sub-structures: the tubule is displayed in blue, the lumen in green and the nuclei in yellow.

**Fig. 4.**
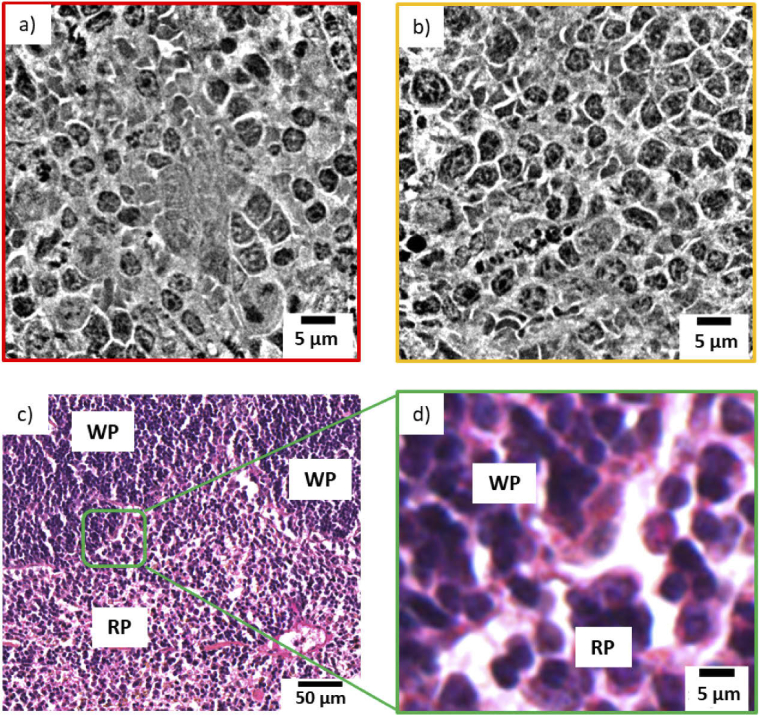
(a) Reconstructed tomographic slice showing the cellular arrangement in the red pulp area of the spleen. (b) Reconstructed tomographic slice representing the cellular arrangement in the white pulp area of the spleen. (c) Histological image showing the cellular arrangement at the interface between red pulp (RP) and white pulp (WP). (d) Magnified imaged showing the cell nuclei belonging to the red pulp (RP) and to the white pulp (WP).

**Fig. 5.**
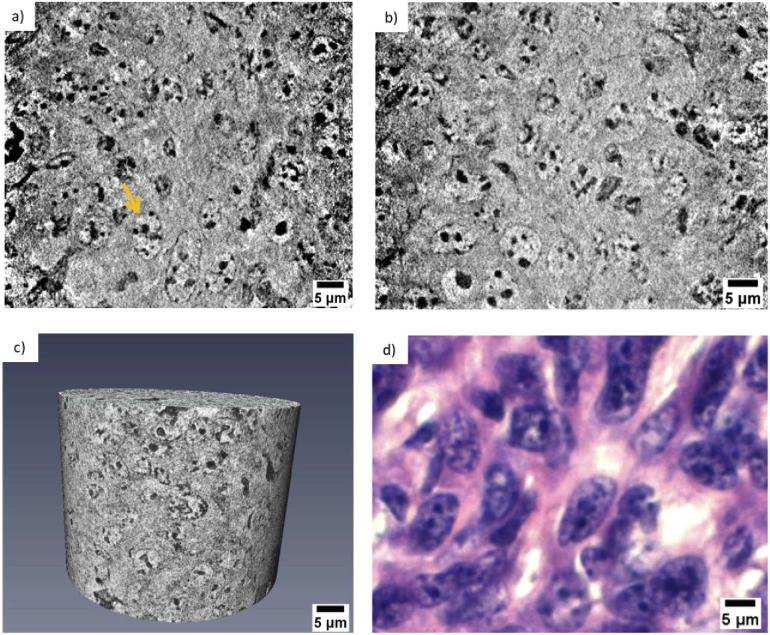
(a) Minimum intensity projection image of 14 reconstructed nanotomography slices. The image shows the chaotic organization of tumor cells surrounding a typical healthy mouse cell, whose nucleus is pointed out by a yellow arrow. (b) Minimum intensity projection image of 14 nanotomography slices selected at another depth of the reconstructed dataset. (c) 3D rendering of a volume of interest with 51 µm in height. (d) Histological image of the same tumor tissue.

The tubule perimeter is marked with a blue dashed line in Fig. 2(b), while the lumen is highlighted by a green dashed line. Structures like the ones indicated by the yellow dashed line are the cellular nuclei. Even black sub-structures are observed inside. These are the nucleoli (typically of 1 µm diameter) and are pointed out by an orange arrow. Outside the tubule, the two red arrows indicate a glomerulus. Figure 2(c) is another nanotomographic insight of the tubule cells distribution in the kidney. The contrast is sufficient for recognizing morphological differences among the tubules. For instance, the central tubule is spherical with a large lumen, while the surrounding tubules have a more elongated shape and a differently structured lumen. Very likely, these structures are the distal and proximal tubule cells in the outer part of the kidney. Both of these types of cell structures are present in the kidney cortex, the anatomical region where this sample was derived. Typically, the proximal tubules have a brush border of microvilli that allows to distinguish them from the distal tubules [45–47]. This feature was not discriminated with our instrument, being in the size range of 100 - 200 nm in length and 50 nm in diameter [48], while it can be easily determined by histology and TEM. However, the distal tubules are known to be small (typically 30 - 40 µm in diameter) and to have a round and large lumen, while the proximal tubules are elongated (typically 60 µm in diameter) with an uneven lumen. As mentioned above, these features are visible in Fig. 2(c) and are even more emphasized in Fig. 2(d), where a distal tubule (blue dashed line) appears to be surrounded by three proximal tubules (purple arrows). Some cell boundaries are visible in the reconstructed slice (Fig. 2(c)) but these very fine structures appear to be obscured in the minimum projection image (Fig. 2(d)). An improvement of the contrast to noise of these structures might be obtained using an optimized denoising algorithm prior to image reconstruction. Figure 2(d) presents the projection of the minimum intensity pixel values of 11 nanotomography slices with similar features. This rendering method virtually increases the thickness of the nanotomography slices and facilitates the comparison with histology [17]. The images obtained by nanotomography can be directly compared with histology. The histological analysis was performed on the same mouse kidney after the nanotomography scan, as it requires sectioning and staining of the sample. A histological image representative of the kidney cortex anatomy is illustrated in Fig. 2(e). A higher magnification view of the region within the black rectangle is displayed in Fig. 2(f) allowing a better comparison with the data obtained by nanotomography. Figure 2(e) shows a mixture of tubule cells (proximal and distal tubule cells) and glomeruli. The tubules are labelled in purple (eosin staining) and the nuclei appear violet in color due to the used staining reagents (hematoxylin staining), while the lumen is visualized in white. In contrast to histology, X-ray nanotomography offers the advantage of handling 3D information. Figure 3(a) presents a 3D rendering of the tomographic images acquired from the kidney tissue. The volume of interest is 73 µm in height and the available field of view allows the visualization of several tubule cells. The clear distinction of the borders of the tubule structures allowed mapping their 3D distribution, organization and packing. Figure 3(b) shows a thinner section (18.6 µm in thickness) and a segmented tubular structure is overlaid in color. Thanks to the differences in contrast, ultra- cellular components can be segmented within the tubule. Figure 3(c) shows its 3D rendering where the tubule is displayed in blue, the lumen in green and the nuclei in yellow. Data segmentation is an important achievement for accessing statistical information, such as average diameter and volume of the labeled structures. Figure 4(a) and 4(b) show two virtual slices from the tomographic reconstruction of a mouse spleen sample. The investigated sample was selected at the interface between the red pulp and the white pulp, which are the main constituent matters of the spleen. The red pulp is characterized by a low cell density, while the white pulp exhibits a higher cell density. As indicated in Fig. 4(a) and 4(b), the X-ray method allowed the visualization of the nuclei belonging to multiple cells presented in the imaged ROIs. Similar to Fig. 2(b), the nuclei are the round tiny structures (typical size from 3 to 10 µm) and the nucleoli (central black spots inside the nuclei, typical size 1 µm) are also resolved. Figure 4(a) shows a lower distribution of cells in the cytoplasm and would correspond to a magnified area of the red pulp region. On the other hand, Fig. 4(b) shows a higher concentration of cells and would correspond to a magnified area of the white pulp region. The histological image in Fig. 4(c) is representative of the cellular distribution at the interface between red pulp and white pulp in the same mouse spleen sample. Figure 4(d) is a magnified region obtained from the histological image illustrated in Fig. 4(c). The staining protocol allowed the nuclei visualization in purple color with a higher concentration of cells detected in the white pulp rather than in the red pulp. Thus, the nanotomography results appear to be in good agreement with histology. Similarly, the cellular distribution in a mouse mammalian carcinoma was studied by nanotomography. This tumor type from mice origin is often selected for its huge capacity to accumulate any drugs after intravenous administration, since it has been reported to have an enhanced permeability and retention effect [49]. Figure 5(a) and 5(b) display two minimum intensity projection images selected from the nanotomography dataset. As it is expected for tumor tissues, Fig. 5 displays a chaotic arrangement of the cells with nuclei showing different structural features. The nucleus, indicated by the yellow arrow in Fig. 5(a), is clearly visualized with several black spots (the nucleoli) typically observed in mice cells. Figure 5(c) visualizes a 3D rendering of a volume of interest with 51 µm in height, selected from the nanotomography dataset. From this 3D representation, it is also possible to distinguish these morphological features of the cells composing the tumor tissue. Finally, Fig. 5(d) is a histological image obtained from this same tumor sample. Cell nuclei are labelled with a purple color and nucleolus are displayed in dark blue. Substantial similarities could be detected comparing the histological images with the tomographic ones, confirming the previous analysis.

## 4. Conclusion and outlook

**Fig. 6.**
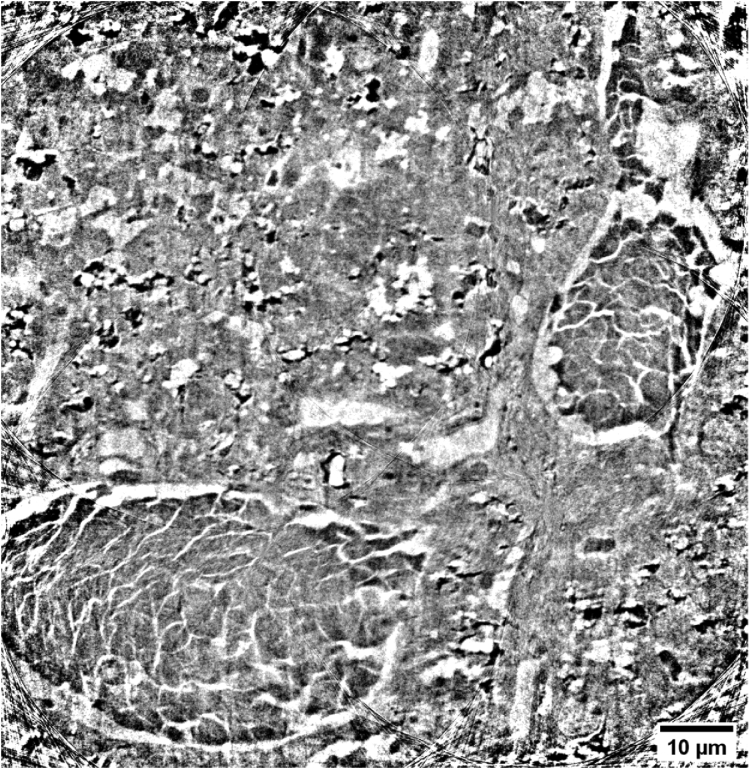
Slice with enlarged FoV obtained by stitching four reconstructed nanotomographic slices.

In this study, we have demonstrated that ROI tomography combined with Zernike phase contrast large field of view microscopy is able to produce high-quality 3D images of different types of mice tissue without staining, labelling, sectioning or FIB sample preparation. The presented nano-imaging approach provided reliable 3D investigations of complex biological specimens, such as mouse organ tissues. As a proof of concept, we acquired images of mouse kidney, spleen, and mammalian carcinoma and we have proved that their anatomy can be fully recovered. Without staining or a specific sample preparation, the presented method enabled a high resolution morphological analysis of the tissues at short acquisition times and non-destructively. The quality of the images, in terms of resolution and contrast, allowed the 3D identification and segmentation of the different tissue components. The obtained 3D information can be considered complementary and supportive to well-known microscopy techniques, such as 2D classical histology where small details are quite often irretrievably lost during the sectioning or invisible if the staining method is not appropriate. X-ray nanotomography offers the advantage of observing the 3D spatial organization of the tissues structures from different orientations and in particular to visualize the inter-cell connections. This is not always easy to recognize by standard 2D imaging methods. The 3D knowledge is of primary interest for detecting cell alterations induced by diseases or the evaluation of the tissue response to possible clinical treatments. Thus, the development of X-ray nanotomography may find a wide range of applications. For instance, its results are very attractive for the field of brain imaging, where the 3D information plays a crucial role for understanding the tissue changes in the case of neurodegenerative diseases. In comparison to histology, however, the presented method enables a high-resolution morphological analysis of the tissues at short acquisition times, without staining and non-destructively. Finally, the relatively fast interior tomographic measurement of below 30 min per scan allows imaging multiple ROIs of the same sample, consecutively. Single volumes can be stitched together resulting in larger volumetric datasets. An example of such a stitched volume is displayed in Fig. 6 (Visualization 1). A 2D nanotomography slice obtained from the stitching of 4 reconstructed nanotomography slices is presented. The scans were performed with an overlap of 40 µm at a scan time of 15 min each. The whole volume of 100 x 100 x 74 µm^3^ was performed in 1h. The stitched image shows a final cropped FoV equal to 101.66 µm x 104.40 µm. Even larger FoV can be achieved by collecting other tomographies in a tile sequence. This is an added-value for this microscopy technique that offers the possibility of detecting small structures in a large adjustable volume of interest. Therefore, we believe that this imaging technique, bridging the scale gap between TEM and histology, can support the understanding of the cell arrangement, revealing abnormalities induced by diseases and pathologies and the influence of clinical treatments from a microscopic point of view, covering applications from biology up to tissue engineering.
